# Multiple Functions of Nm23-H1 Are Regulated by Oxido-Reduction System

**DOI:** 10.1371/journal.pone.0007949

**Published:** 2009-11-23

**Authors:** Eunsun Lee, Jaeho Jeong, Sung Eun Kim, Eun Joo Song, Sang Won Kang, Kong-Joo Lee

**Affiliations:** Center for Cell Signaling and Drug Discovery Research, Division of Life and Pharmaceutical Sciences, College of Pharmacy and Department of Bioinspired Science, Ewha Womans University, Seoul, Korea; University of Southampton, United Kingdom

## Abstract

Nucleoside diphosphate kinase (NDPK, Nm23), a housekeeping enzyme, is known to be a multifunctional protein, acting as a metastasis suppressor, transactivation activity on c-myc, and regulating endocytosis. The cellular mechanisms regulating Nm23 functions are poorly understood. In this study, we identified the modifications and interacting proteins of Nm23-H1 in response to oxidative stress. We found that Cys109 in Nm23-H1 is oxidized to various oxidation states including intra- and inter-disulfide crosslinks, glutathionylation, and sulfonic acid formation in response to H_2_O_2_ treatment both *in vivo* and *in vitro*. The cross-linking sites and modifications of oxidized Nm23-H1 were identified by peptide sequencing using UPLC-ESI-q-TOF tandem MS. Glutathionylation and oxidation of Cys109 inhibited the NDPK enzymatic activity of Nm23-H1. We also found that thioredoxin reductase 1 (TrxR1) is an interacting protein of Nm23-H1, and it binds specifically to oxidized Nm23-H1. Oxidized Nm23 is a substrate of NADPH-TrxR1-thioredoxin shuttle system, and the disulfide crosslinking is reversibly reduced and the enzymatic activity is recovered by this system. Oxidation of Cys109 in Nm23-H1 inhibited its metastatic suppressor activity as well as the enzymatic activities. The mutant, Nm23-H1 C109A, retained both the enzymatic and metastasis suppressor activities under oxidative stress. This suggests that key enzymatic and metastasis suppressor functions of Nm23-H1 are regulated by oxido-reduction of its Cys109.

## Introduction

Nucleoside diphosphate kinase (NDPK, Nm23), a housekeeping enzyme, is considered to be a tumor metastasis suppressor [1], [2]. Nm23 is characterized by its reduced expression in melanoma cell lines having high metastatic potential [3]. Overexpression of cellular Nm23-H1 correlated with decreased metastasis potential of breast, melanoma, colon, and oral squamous cells [4]. Also, mutations of Nm23-H1 seen in some cancers including colorectal carcinoma, are associated with increased metastasis [5].

Nm23 belongs to a family of structurally conserved proteins of NDP kinase catalyzing the transfer of terminal phosphate from nucleoside triphosphate (NTP) to nucleoside diphosphate (NDP) [6]. There are eight known *nm23* genes in the human genome, and two of them, Nm23-H1 and Nm23-H2, have been the most widely studied. Nm23-H1 and -H2 are small proteins consisted with152 amino acids, and form homohexamers or heterohexamers [7]. Although they are highly homologous (88% amino acid identity), their cellular functions and localizations are different. Nm23-H1 is a putative metastasis suppressor of some tumor types [3], [6], [8], whereas Nm23-H2 binds to the nuclease-sensitive element of *c-myc* gene promoter, and transactivates its gene expression [9], [10], [11]. Both Nm23-H1 and -H2 proteins are found in the cytoplasm, but Nm23-H2 has also been detected in the nucleus [12], [13].

Studies, performed to understand the molecular mechanism underlying the ability of Nm23-H1, to suppress metastasis, led to following observations. Nm23-H1 regulates some small G-proteins which play important roles in cell migration as a GTPase [14]. Nm23-H1 inhibits MAP kinase pathway by interacting with kinase suppressor of Ras 1 (KSR1) scaffold protein. Nm23-H1 in its function as a protein kinase, forms a complex with KSR1 and phosphorylates it at Ser 392 and Ser 434, which results in blockade of Ras/MAPK pathway [15], [16]. And Nm23-H1 interacts with Tiam1, a specific guanine nucleotide exchange factor (GEF) for Rac1, and down-regulates Tiam1-Rac1 signaling, implying that it affects remodeling of the actin cytoskeleton [17]. Palacios *et al*. demonstrated that constitutively activated ARF6 binds to Nm23-H1 and recruits Nm23-H1 to cell junction [18]. More recently, Nm23-H1 has been shown to interact with Dbl-1, an oncoprotein of the GEF family, thereby inhibiting the suppression of cell motility by Nm23-H1. Moreover, this interaction results in the loss of the ability of the Dbl-1 to function as a GEF for Cdc42 [19]. However, how Nm23s exerts variety of cellular activities and how these activities are regulated, is not well understood.

Post-translational modifications (PTMs) of Nm23 play key roles in many cellular functions and regulatory processes, by influencing cellular localization, protein–protein interactions, and biological activities of cellular proteins. The modifications of Nm23 caused by oxidative stresses were identified as disulfide crosslinking [20]. Cellular Nm23 is cross-linked by disulfide bond in response to H_2_O_2_ treatment and is reversibly reduced by reducing agent dithiothreitol (DTT) or by some unknown cellular mechanism. H_2_O_2_ inactivates NDP kinase activity of Nm23 by producing disulfide bonds which cause the dissociation of native hexameric structure to dimeric form. Intermolecular cross-linking sites between Cys109-Cys109 were identified by mass spectrometry. Even though Cys109 residue is not in the active site, oxido-reduction of this residue can regulate the enzymatic activity and the quaternary structure of Nm23. However, the cellular regulation of Nm23 was not clearly understood.

All organisms possess repair systems for removing damaged/oxidized molecules. Thioredoxin reductase 1 (TrxR1) is a selenium-containing pyridine nucleotide-disulfide oxidoreductase, with mechanistic and sequence identity with glutathione reductases, including a conserved CVNVG redox catalytic site. TrxR catalyses the NADPH-dependent reduction of the redox protein thioredoxin (Trx), as well as of other endogenous and exogenous compounds. TrxRs are also named because of their ability to reduce oxidized Trx [21], [22]. Mammalian Trxs are a family of small (12 kDa) redox proteins that undergo NADPH-dependent reduction mediated by TrxR and which in turn reduce oxidized cysteines on proteins. Trx1 performs many biological functions including supplying reducing equivalents to thioredoxin peroxidases and ribonucleotide reductase, regulating transcription factor activity, and the enzyme activity by heterodimer formation [23]. In this study, we identified the various oxidative modifications of cellular Nm23-H1 using UPLC-ESI-q-TOF tandem MS including S-glutathionylation of Cys109. The increase of protein glutathionylations are believed to be the cellular response to oxidative stress [24]. S-glutathionylation is a reversible post-translational modification and may proceed spontaneously by thiol-disulfide exchange or by reaction of a reduced protein-SH or GSH with an oxidized sulfhydryl [25]. And S-glutathionylation has been shown to be essential for the function of several proteins such as Ras, NF-1, c-jun, p50/NF-κB, actin and IκB kinase subunit β (IKKβ) [26]–[31]. This modification inhibits NDPK activity of Nm23-H1. We also found TrxR to be an interacting protein of oxidized Nm23-H1 and that oxidized Nm23-H1 can be repaired by NADPH-TrxR-Trx system. This suggests that Nm23-H1 can be a key molecule modified and regulated by ROS via NADPH-TrxR-Trx system. These results suggest that the biological function of Nm23-H1, as a house keeping enzyme, and as a tumor metastasis suppressor, is regulated by oxido-reduction of Nm23-H1 at a cysteine residue, not at its active site.

## Materials and Methods

### Materials

Monoclonal anti-Flag antibody (M2) was purchased from Sigma (St. Louis, MO, USA), monoclonal anti-Nm23-H1 antibody and anti-tubulin from Santa Cruz Biotechnology (Santa Cruz, CA, USA), horseradish peroxidase conjugated goat-anti-mouse IgG and goat anti rabbit IgG from Bio-Rad Laboratories (Richmond, CA, USA), polyclonal anti-TrxR1 and anti-Trx1 antibodies from Ab Frontier (Seoul, Korea). Polyclonal anti-serum of Nm23-H2 was raised in rabbits using purified recombinant Nm23-H2 as the immunogen. Protein G sepharose was purchased from GE healthcare (Giles, United Kingdom). Other biochemicals including 4-hydroxy-α-cyano-cinnamic acid (HCCA), 3,5-dimethoxy-4-hydroxy cinnamic acid (sinapinic acid), trifluoroacetic acid (TFA), sodium bicarbonate, and ATP-agarose were purchased from Sigma (St. Louis, MO, USA). PEI cellulose TLC plates were purchased from Altech (Deerfield, IL, USA), sequencing grade trypsin (Promega). Radioisotope [γ-^32^P]ATP was obtained from DuPont NEN™ (Boston, MA, USA).

### Plasmids and protein purification

Expression plasmids pET3c containing nm23-H1 and nm23-H2 were provided by Dr. P. S. Steeg at NCI (USA). For expression in mammalian cells nm23-H1 or nm23-H2 was cloned into Flag vector. *E. coli* strain BL21 (DE3) was used for protein expression. Recombinant NDPK-A (Nm23-H1) and NDPK-B (Nm23-H2) were purified as described previously [32]. Cytosolic fraction of *E. coli* strains BL21 (DE3) transformed with pET-3c expression plasmids containing nm23-H1 coding region were obtained after inducing the expression of each protein with 0.2 mM IPTG with the method previously described. Each cytosolic fraction was applied to 2∼4 mL of ATP-sepharose column equilibrated with Buffer A (20 mM Tris-acetate, 20 mM NaCl, 0.1 mM EDTA, 3 mM MgCl_2_, pH 7.4) at a flow rate of 3 mL/min. The column was then washed with buffer A and then with Buffer A containing 0.25 M NaCl to remove nonspecifically binding proteins. Then NDPK was eluted with Buffer A containing 1 mM ATP [32]. Rat TrxR1 and recombinant rat Trx1 were purified as described [33].

### Cell culture

HeLa (human epithelial carcinoma) cells, and HEK293T (human embryonic kidney epithelial) cells, were grown and maintained in high glucose Dulbecco's modified Eagle's medium (DMEM) supplemented with 10% fetal bovine serum (FBS), 100 µg/mL streptomycin, 100 units/mL penicillin G, 3.75 µg/mL sodium bicarbonate and 0.11 µg/mL sodium pyruvate at 37°C and 5% CO_2_. MDA-MB-231 cells (ATCC, VA, USA) were grown in Eagle's minimum essential medium (EMEM) and MCF-7 cells (KCLB, KOREA) in RPMI 1640 supplemented with 10% fetal bovine serum (FBS), 100 µg/mL streptomycin, 100 units/mL penicillin G at 37°C in an atmosphere of 5% CO_2_-95% air.

### Transient transfection and H_2_O_2_ treatment

HEK293T cells were transfected with expression plasmids using the calcium phosphate precipitation method. Cells were seeded in plates a day before transfection at the density of 2.5×10^5^ cells and transiently transfected with expression plasmids. For 35 mm dishes, 1.5∼2 µg of plasmid DNA suspended in 131.4 µL of H_2_O were mixed with 18.6 µL of 2 M CaCl_2_ and immediately added to 150 µL of 2 x HBSS (50 mM HEPES, pH 7.05, 10 mM KCl, 12 mM glucose, 280 mM NaCl, 1.5 mM Na_2_HPO_4_) and then to the cells 1 h after adding the fresh medium to the cells. HeLa cells were seeded in 35 mm plates at 1.25×10^5^ cells and transfected with maximum 2 µg of plasmid by using Lipofectamine (GIBCO BRL and Life Technologies, Rockville, MD) according to manufacturer's protocol. MDA-MB-231 cells were transfected with expression plasmids using TransIT®-LT1 transfection reagent (Mirus, WI, USA). Cells were seeded in 35 mm plates for a day before transfection at the density of 2.5×10^5^ cells and transiently transfected with 2.5 µg of expression plasmids and 7.5 µL TransIT®-LT1 transfection reagent in Opti-MEM solution. After 6 h of incubation at 37°C/5% CO_2_, the transfected cells were returned to medium containing 10% fetal bovine serum, cultured for additional 24 h and subsequently subjected to hydrogen peroxide treatment.

### RNA interference of Nm23-H1

Constructs were obtained from the pSUPER.retro.puro plasmid (a generous gift from Dr. Lu Chen) using specific oligonucleotides of the Nm23-H1 sequence, indicated by capital letter, gatccccCCGCCTTGTTGGTCTGAAAttcaagagaTTTCAGACCAACAAGGCGGtttta (forward), and a gctaaaaaCCGCCTTGTTGGTCTGAAAtctcttgaaTTTCAGACCAACAAGGCGGggg (reverse). Oligonucleotides were obtained from Genotech (Seoul, Korea) and annealed to obtain hairpin structures. And they were cloned HindIII sites of pSUPER.retro.puro plasmid. Three plasmids, a pSUPER.retro.puro plasmid, a VSVG expression vector, a gag-pol expression vector, were co-transfected into 293T cells at a 1∶1∶1 molar ratio by calcium phosphate method. The cultured supernatant containing viral vector particles was harvested 48 h later, filtered with a 0.45 µm membrane filter (Nalgene, NY, USA). Retroviruses were added to MCF-7 cells with 4 µg/µL polybrene and medium. After 48 h, second infection was done as described above. The selection was started after 3 days, adding 500 µg/mL G418 (Sigma, MO, USA) to the culture medium for 10 days. Knock-down of Nm23-H1 was detected with western analysis.

### NDP kinase assay

NDP kinase enzymatic activity of NDPK-A (Nm23-H1) was measured as previously described [20]. Enzymes were incubated in 20 µL total volume of reaction buffer B containing 20 mM HEPES (pH 7.4), 0.1 mM ATP and 1 mM UDP as substrate, 0.1 µCi [γ-^32^P]ATP and 3 mM MgCl_2_ for 10 min at 37°C. The reaction was stopped by adding gel sample buffer containing 125 mM Tris base, 2.3% SDS and 10% glycerol. Aliquots were loaded onto PEI cellulose TLC plates and developed in a solution of 0.75 M KH_2_PO_4_ (pH 3.6). Dried TLC plates were exposed for autoradiogram and the formation of [γ-^32^P]UTP was quantified with Fujiphotofilm BAS2000 (Tokyo, Japan). All measurements were made in triplicate.

### NADPH-TrxR-Trx system assay

TrxR assay was performed in 50 mM sodium phosphate, pH 7.0, 50 mM KCl, 1 mM EDTA, 100 µM Nm23-H1, 200 µM NADPH, 80 nM rat TrxR1, 50 µM Trx1 and the absorbance decrease of NADPH was monitored at 340 nm for 5 min by spectrophotometer [33], [34].

### Immunoblotting and immunoprecipitation

Proteins separated by SDS-PAGE were immediately transferred onto PVDF membrane for 1 h with 50 mA. Membrane was blocked with 3% bovine serum albumin (BSA) in PBS containing 0.1% Tween 20 for 2 h at room temperature and sequentially incubated with each antibody diluted 1∶1000 in PBS containing 3% BSA for 2 h at room temperature. After washing three times with PBS containing 0.1% Tween 20 for 10 min, membrane was incubated with second antibody, goat anti-mouse antibody or goat anti-rabbit antibody, diluted 1∶2000 in PBS containing 0.1% Tween 20, then washed with PBS containing 0.1% Tween 20 for 10 min three times. Detection of the immunocomplexes was performed using West-oneTM (iNtRON biotechnology, KOREA) and LAS-3000s (Fuji photo film, Tokyo, Japan).

The cell pellet was lysed with modified radio-immunoprecipitation assay buffer containing protease inhibitors (RIPA: 50 mM Tris-HCl [pH 7.8], 150 mM NaCl, 5 mM EDTA, 15 mM MgCl_2_, 0.5% Nonidet P-40, 0.3% Triton X-100, 1∶200-diluted protease inhibitor cocktail, 5 mM Na_3_VO_4_, 20 mM NEM) for 30 min on ice. The lysates were centrifuged at 12,000 rpm for 15 min and the supernatant was incubated for 3 h at 4°C with anti-Flag antibody cross linked to protein G sepharose beads or anti-Nm23-H1 crosslinking protein G sepharose beads. The beads were washed three times with 1 mL of RIPA buffer containing 0.5% Nonidet P-40 and 0.3% Triton X-100 and three times with RIPA buffer without detergent. Proteins were solubilized with non-reducing gel sample buffer, separated on SDS-PAGE under non-reducing condition, and then detected with coomassie staining, silver staining, or western analysis after transferring onto PVDF membrane.

### *In vitro* cell migration assay

This was performed using a 24-well chemotaxis chamber (transwell, Costar). The upper chamber of transwell was coated with 50 mg Matrigel TM basement membrane matrix (BD Biosciences) for 1 h at 37°C and then placed into 24 well chambers. The lower chamber was filled with media containing 10% FBS. The cells were seeded in the upper chamber (1.5×10^4^ cells/150 µL) and then incubated for 24 h at 37°C. The migrating cells were stained with crystal violet (0.5% w/v crystal violet, 25% methanol) and counted at 100-fold magnification using a microscope.

### Sample preparation for mass spectrometry analysis

The proteins obtained from immunoprecipitated complex were separated on gel electrophoresis under non-reducing condition and detected with silver, or coomassie blue staining. The gel bands were excised with a scalpel, destained by each destaining reagent (30 mM potassium ferricyanide/100 mM sodium thiosulfate, or 25 mM ammonium bicarbonate/60% ACN) and washed to remove destaining reagent. The pH was adjusted to 8.0 with 200 mM ammonium bicarbonate to facilitate trypsin digestion. The gels were dehydrated by addition of acetonitrile, rehydrated by adding 10∼20 µL of 25 mM NH_4_HCO_3_ with 20 ng/µL of sequencing grade trypsin (Promega Co.), and incubated at 37°C for 15∼17 h. Peptides were extracted with 30 µL of solution containing from 60% ACN/0.1% TFA to 100% ACN. The extracts was evaporated to dryness in SpeedVac and dissolved in 10% ACN/0.1% formic acid to facilitate electrospray.

### Peptide sequencing by liquid chromatography and mass spectrometry

Peptides were analyzed by nanoAcquity™ UPLC™/ESI/MS (SYNAPT™ HDMS™, Waters Co. UK). Peptides were separated by using a C18 reversed-phase 75 µm i.d.×200 mm analytical column (1.7 µm particle size, BEH130 C18, Waters) with an integrated electrospray ionization PicoTip™ (±10 µm, New Objective, USA). 5 µL of peptide mixtures were dissolved in buffer A (Water/formic acid; 100∶0.1, v/v), injected on a column and eluted by a linear gradient of 5-60% buffer B (ACN/formic acid; 100∶0.1, v/v) over 120 min. Samples were desalted on line prior to separation using trap column (i.d. 180 µm×20 mm, Symmetry® C18, Waters) cartridge. Initially, the flow rate was set to 300 nL/min and the capillary voltage (2.8 keV) was applied to the UPLC™ mobile phase before spray. Chromatography was performed on line to SYNAPT™ HDMS™. The mass spectrometer was programmed to record scan cycles composed of one MS scan followed by MSMS scans of the 3∼4 most abundant ions in each MS scan. MS parameters for efficient data-dependent acquisition were intensity (>10), number of components (3∼4) to be switched from MS to MS/MS analysis. In the first run analysis, the 4 most abundant precursors were selected for MS/MS analysis. Following positive identification, all identified peptides from database search (Mascot) were non-redundantly excluded in the next run analysis until almost full sequence coverage was obtained [35], [36].

Database analysis was carried out using the database search program Mascot (global search engine), ProteinLynx Global SERVER (PLGS) 2.3 (Waters Co., UK) and MOD^i^ (Korea, http://prix.uos.ac.kr/modi/). Almost full sequence coverage was provided on selective exclusion monitoring. MS/MS spectra were matched against amino acid sequences in NCBI (USA) and SwissProt. Large number and types of potential PTMs were considered. All reported assignments were verified by automatic and manual interpretation of spectra from Mascot and Proteinlynx search engine and MOD^i^ in a blind mode [37], [38].

## Results

### Cys109 in cellular Nm23-H1 is oxidatively modified

**Figure 1:**
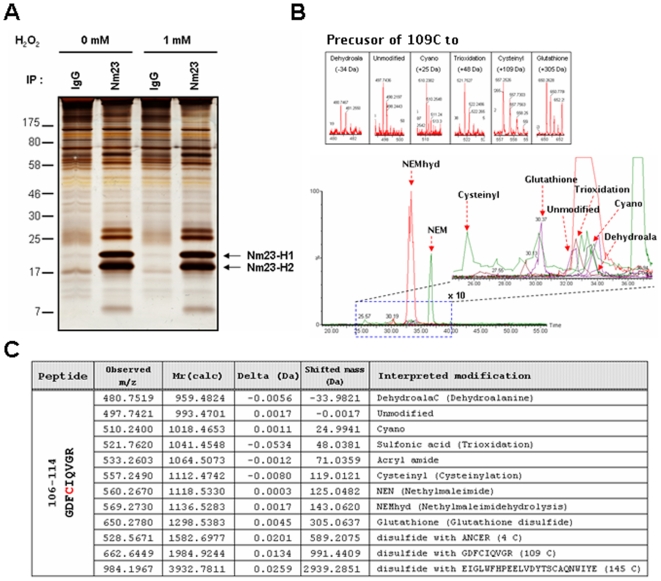
Post-translational modifications of cellular Nm23-H1 in MCF-7 breast cancer cells. (A) MCF-7 breast cancer cells, non-invasive cell lines highly expressing Nm23-H1, were exposed to control and 1 mM H_2_O_2_ for 1 h at 37°C. Endogenous Nm23-H1 and Nm23-H2 were purified by immunoprecipitation using anti-Nm23-H1 antibody. These immuno-complexes were separated on 12% SDS-PAGE under non-reducing condition and detected with silver-staining. (B) Post-translational modifications in active cysteine containing tryptic peptide of Nm23-H1, ^106^GDFCIQVGR^114^ (993.4701 Da) were identified by nanoLC-ESI-q-TOF tandem MS. Bottom panel is a total ion chromatogram containing various modifications of peptide ^106^GDFCIQVGR^114^, and dotted box of this chromatogram was magnified to right panel. Precursor ion of each modified peptide was shown in each box. (C) Table presents the predicted and detected molecular mass and interpreted modifications at Cys-109.

**Figure 2:**
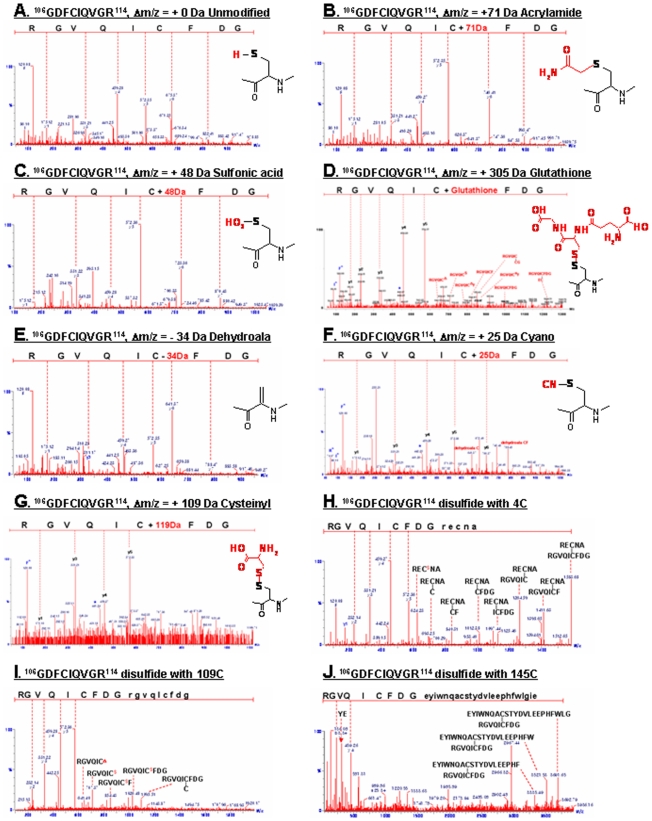
Peptide sequencing using MS/MS analysis of tryptic peptide from modified peptide at Cys-109. (A) Unmodified peptide ^106^GDFCIQVGR^114^; (B) Acrylamide adduct of free SH; (C) Oxidized C109 to sulfonic acid (-SO_3_H), +48 Da at C109; (D) Glutathionylated C109; (E) Dehydroalanine (Δm/z = −33.9821 Da); (F) Formation of Cys-S-CN, +25 Da; (G) Cysteinylation (^109^CysS-SCys, Δm/z = +119.0 Da); (H) inter-disulfide C109; (I,J) Intra-disulfide bond with Cys4 peptide (^109^CysS-S-AN^4^CER, Δm/z = +589.2075 Da) and C145 (^109^CysS-S-ELGLWFHPEELVDYTS^145^CAQNWIYE, Δm/z = +2939.2851 Da).

To determine the cellular modifications of endogenous Nm23-H1 and -H2 by oxidative stress, cellular Nm23-H1 was co-purified with Nm23-H2 by immunoprecipitation using Nm23-H1 antibody (Figure 1A) and various PTMs were analyzed using nanoAcquity™ UPLC™/ESI/q-TOF tandem MS as described in methodology. MCF-7 breast cancer cells, non-invasive cell lines highly expressing Nm23, were exposed to control HBSS or 1 mM H_2_O_2_ for 1 h at 37°C. The immuno-complexes were divided into 2 fractions. One fraction was separated on 12% non-reducing SDS-PAGE under non-reducing condition and detected by silver-staining (Figure 1A). Another fraction of samples was prepared for MS analysis without any staining after separation on 12% SDS-PAGE under non-reducing condition. Presumed Nm23 bands were excised with a scalpel on gel and confirmed as Nm23-H1 and Nm23-H2 by peptide sequencing with nanoAcquity™ UPLC™/ESI/q-TOF tandem MS after trypsin digestion (data not shown). We examined the modification site and status of the modified peptides by peptide sequencing with MS/MS, employing SEMSA technology [36] and MOD^i^ algorithm for PTM detection [37], [38]. As shown in Figure 1B and 1C, we detected various modifications at Cys109 of peptide ^106^GDFCIQVGR^114^ (993.4701 Da). Most of the free sulfhydryl of Cys109 was easily labeled with alkylating agent NEM or by generating acrylamide adduct in SDS-PAGE (Δm/z = +71.0359 Da). But small fractions of Cys109 were oxidiatively modified to various states which included: sulfonic acid modification (^109^CysSO_3_H, Δm/z = +43.038 Da), cyanylation (^109^CysSCN, Δm/z = +24.9941 Da), glutathionylation (^109^CysS-S-glutathione(ECG), Δm/z = +305.0637 Da), cysteinylation (^109^CysS-SCys, Δm/z = +119.0121 Da), dehydroalanine (Δm/z = −33.9821 Da), and disulfide bond formation with other cysteine residues (^109^CysS-S-AN^4^CER, Δm/z = +589.2075 Da: ^109^CysS-S-ELGLWFHPEELVDYTS^145^CAQNWIYE, Δm/z = +2939.2851 Da). Although these oxidative modifications constituted a minor fraction, compared to the NEM labeling, the precursor ion of each oxidative population was clearly identified in Figure 1B. Furthermore, these were confirmed in MS/MS spectra for peptide sequencing in Figure 2. These results indicate that cellular Cys109 of Nm23-H1 is oxidized to various ways. Intriguingly, glutathionylation and sulfonic acid modification at Cys109 were discernibly increased in Nm23-H1 treated with H_2_O_2_ than control, and semi-quantitative analyses of modified peptides were performed by quantifying the precursor ion intensities in LC chromatograms (Figure S1). These results suggest that Cys109 plays a crucial role in response to oxidative stress through glutathionylation.

### Glutathionylation of Nm23-H1 occurred at Cys109 residue

**Figure 3:**
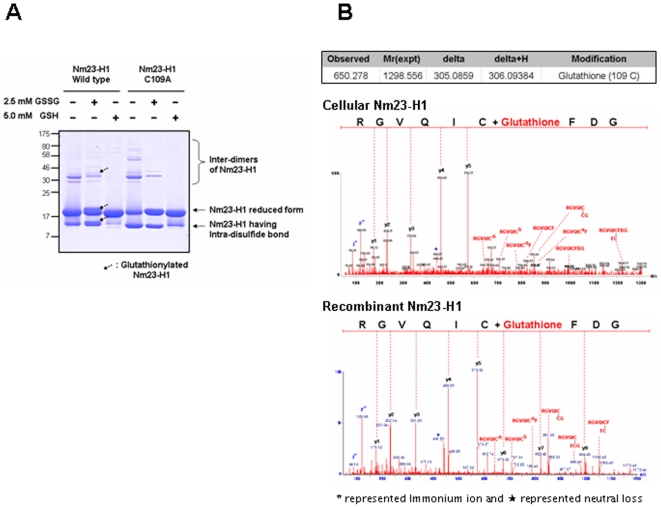
MS/MS spectra studies of tryptic peptide reveal glutathionylation of Nm23-H1. (A) Purified recombinant proteins, wild type Nm23-H1 and C109A mutant, were treated with and without 2.5 mM oxidized glutathione (GSSG), 5 mM reduced glutathione (GSH) at 37°C for 1 h. Each sample was separated on 12% SDS-PAGE under non-reducing condition and detected with coomassie blue staining. Only glutathionylation of wild Nm23-H1, not C109A mutant, was easily observed. (B) Tandem MS spectra of glutathionylated cellular and recombinant Nm23-H1 were compared. Both mass spectra profile was identical, only abundance of glutathionylated peptide is higher in recombinant protein.

To confirm the glutathionylation of Cys109 in Nm23-H1, the glutathionylations of recombinant Nm23-H1s were examined by treating them with glutathione. Recombinant proteins of wild Nm23-H1 and C109A mutant were treated with 2.5 mM oxidized glutathione (GSSG) or 5 mM reduced glutathione (GSH) for 1 h at 37°C. Each sample was separated on 12% SDS-PAGE under non-reducing condition and detected with coomassie blue staining (Figure 3A). The original band of wild type Nm23-H1 with GSSG shifted to higher molecular weight. However, GSSG treatment did not affect the band profiles of C109A mutant. The shifted bands from wild type Nm23 were identified as products of glutathionylation at Cys109 by nanoAcquity™ UPLC™/ESI-q-TOF tandem MS. Tandem MS spectra of glutathionylated peptides (^106^GDFCIQVGR^114^) from cellular and recombinant Nm23-H1 were compared in Figure 3B. The patterns of MS/MS spectra were typical and quite similar to the disulfide bond fragmentation even though the intensity of cellular Nm23 was lower than that of the recombinant one. MS/MS fragmentations of glutathionylated peptide were assigned in Figure S2. These results demonstrate that Cys109 of Nm23-H1 is easily glutathionylated by oxidized glutathione.

### Glutathionylated Nm23-H1 inhibits NDPK enzymatic activity

**Figure 4:**
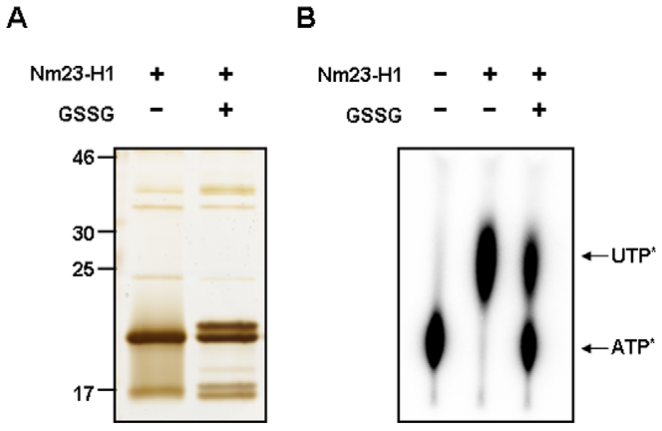
Glutathionylation of Nm23-H1 abolishes NDP kinase enzymatic activity. (A) Recombinant wild type Nm23-H1 was treated with 5.0 mM glutathione for 30 min at 37°C. Each sample was separated on 12% SDS-PAGE under non-reducing condition and detected with silver staining. (B) NDP kinase activity of each sample was measured as described in Experimental procedure. Glutathionylated fraction of sample lost their NDP kinase activity.

To determine the effect of glutathionylation on the biological activity of Nm23-H1, we examined the NDPK activity of glutathionylated Nm23-H1. Recombinant Nm23-H1 was treated with 5.0 mM oxidized glutathione (GSSG) for 30 min at 37°C. The product was separated by 12% SDS-PAGE gel under non-reducing conditions and silver stained. Half of Nm23-H1 was glutathionylated (as shown in Figure 4A), furthermore, the NDP kinase activity of Nm23 disappeared with glutathionylation (Figure 4B). These results suggest that the glutathionylation of Cys109 of Nm23-H1 alters the structure of its active site and inhibits their enzymatic activity.

### Oxidized Nm23 interacts with thioredoxin reductase in vivo

**Figure 5:**
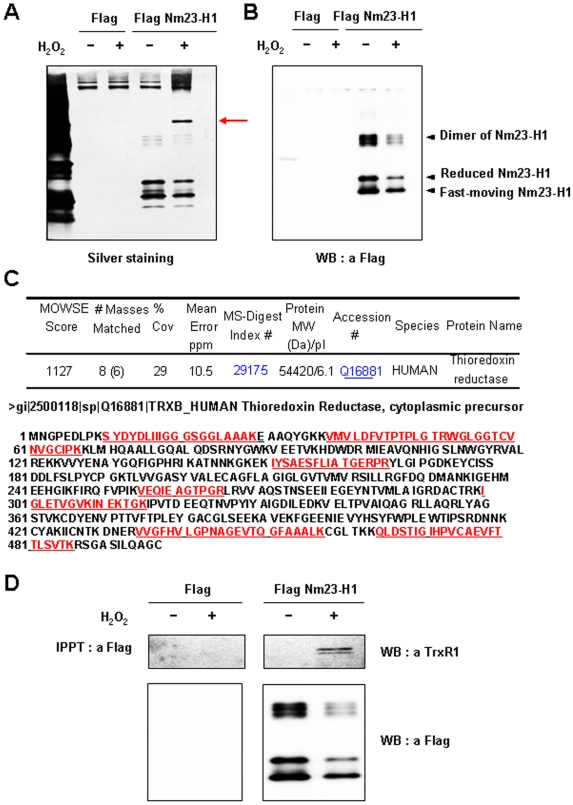
Oxidized Nm23-H1 interacts with thioredoxin reductase 1. (A,B) Hela cells transiently transfected with Flag-Nm23-H1 were exposed to 5 mM H_2_O_2_ for 1 h at 37°C and recovered for 6 h without H_2_O_2_. Endogenous Nm23-H1s were purified by immunoprecipitation using anti-Flag antibody. These immuno-complexes were separated on 12% SDS-PAGE under non-reducing condition and detected with silver-staining (A) and with western analysis using anti-Flag antibody (B). (C) Protein interacting with oxidized Nm23 as indicated arrow in Figure 4A was identified with MALDI-TOF MS as thioredoxin reductase (TrxR). (D) Thioredoxin reductase interacting with oxidized Nm23-H1 was confirmed by western analysis using anti-TrxR antibody.

We attempted to identify the proteins interacting with oxidized Nm23-H1 in order to understand how oxidized Nm23-H1 functions *in vivo*. Hela cells transiently overexpressing Flag-Nm23-H1 were exposed to 5 mM H_2_O_2_ for 1 h and let stand for 6 h without H_2_O_2_. Hela cell lysates were immunoprecipitated with monoclonal anti-Flag M2 agarose cross-linked affinity beads, and the immunoprecipitated complexes were separated on one dimensional gel electrophoresis and detected by silver staining (Figure 5A) and by Western analysis using anti-Flag antibody (Figure 5B). Flag-Nm23s were detected at 17 kDa and 34 kDa with silver staining (Figure 5A) and confirmed by Western blotting (Figure 5B). In addition to Nm23-H1, we detected a new band in the co-immunoprecipitates with oxidized Flag-Nm23-H1, but not with control Flag and control Flag-Nm23-H1 (Figure 5A). This band was identified as thioredoxin reductase 1 (TrxR1, NCBI accession number 2500118) (Figure 5C), after subjecting it to in-gel digestion with trypsin and MALDI-TOF MS and nanoLC-ESI-q-TOF MS/MS, analysis. The identity of this protein binding to oxidized Nm23 as thioredoxin reductase 1 (TrxR1) was confirmed by Western analysis using anti-TrxR1 antibody (Figure 5D). Thus oxidized Nm23-H1 seems to specifically interact with TrxR1 *in vivo*.

### Oxidized Nm23 is a substrate of thioredoxin reductase 1- thioredoxin-NADPH system

**Figure 6:**
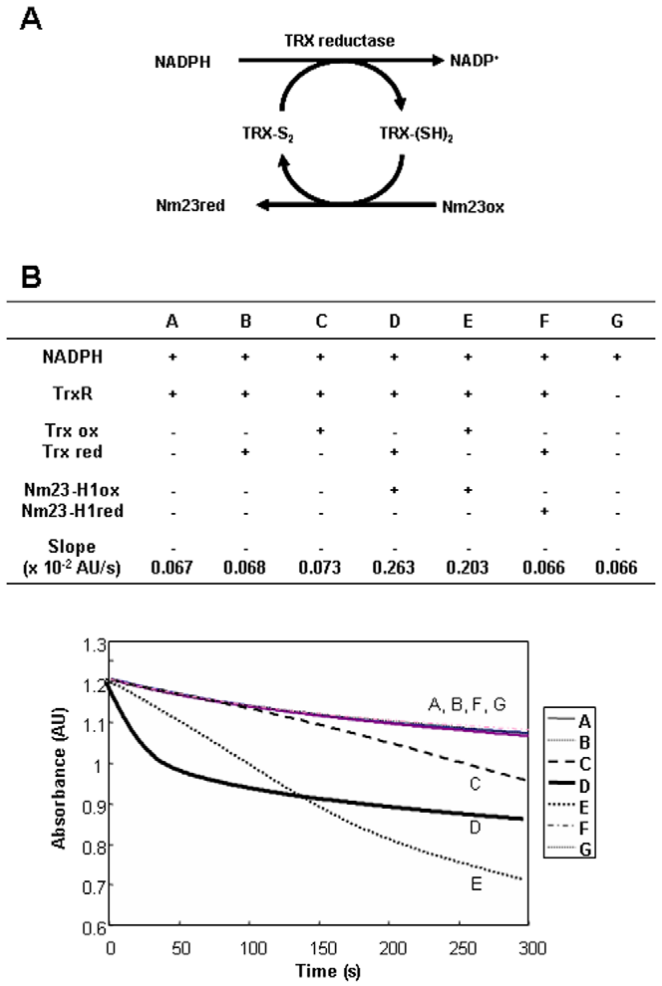
Oxidized Nm23 is a substrate of NADPH-TrxR-Trx system. (A) Suggested scheme of oxido-reduction regulation of Nm23-H1 by TrxR-Trx-NADPH system. (B) TrxR assay was performed in solution containing 50 mM sodium phosphate, pH 7.0, 50 mM KCl, 1 mM EDTA, 100 µM Nm23-H1, 200 µM NADPH, 80 µM rat TrxR1, 50 µM Trx1, and monitored the absorbance change at 340 nm. Absorbance decreases by conversion of NADPH to NADP were measured as TR activity. It turns out that oxidized Nm23-H1 is a substrate of TrxR-Trx-NADPH system.

Thioredoxin reductase 1 (TrxR1) is known enzyme to catalyze the reduction of oxidized thioredoxin (Trx), by transferring electrons from NADPH to the active-site disulfide of oxidized Trx. We suggest that oxidized Nm23-H1 may be a substrate of TrxR1 or Trx (Figure 6A). To examine whether oxidized Nm23-H1 regulates the TrxR1 system, we monitored TrxR1 activity by measuring the conversion rate of NADPH to NADP in reaction mixtures containing 50 mM sodium phosphate, pH 7.0, 50 mM KCl, 1 mM EDTA, 200 µM NADPH, and varying combinations of 80 nM rat TrxR1, 50 µM Trx and 100 µM Nm23-H1. The oxidation kinetics from NADPH to NADP^+^ was determined by measuring absorbance changes at 340 nm in various combinations of reaction components as shown (Figure 6B). The changes of A_340_ in control (A, G) and reduced Trx (B) by NADPH and TrxR1 were about –0.067, and in oxidized Trx (C) −0,073, which indicates the reduction of oxidized Trx through NADPH and TrxR1. When we added oxidized Nm23-H1 (D, E) to the mixture of NADPH, TrxR1 and reduced (D) or oxidized Trx (E), the absorbance changes observed were A_340_, −0.263 and −0.203 respectively. The velocity of the reaction in sample D using reduced Trx, is markedly faster in sample E using oxidized Trx, confirming that oxidized Nm23 is a substrate of Trx coupled with reduced Trx. On the other hand, reduced Nm23-H1 (F) cannot oxidize NADPH, indicating that oxidized Nm23 can be specifically reduced in NADPH-TrxR-Trx system. We examined the possibility of Nm23 as a direct substrate of TrxR as like Trx. When oxidized and reduced Nm23s were incubated with NADPH and TrxR without Trx, the absorbance decrease was negligible as with the control (Figure S3E and S3F). This suggests that Nm23 is not a direct substrate of TrxR, and that Trx is necessary for reducing oxidized Nm23-H1. Thus it appears that oxidized Nm23 can be a substrate of Trx in NADPH-TrxR-Trx system and that oxidized Nm23-H1 can be reduced by the oxidative conversion of NADPH to NADP^+^ by electron donation.

**Figure 7:**
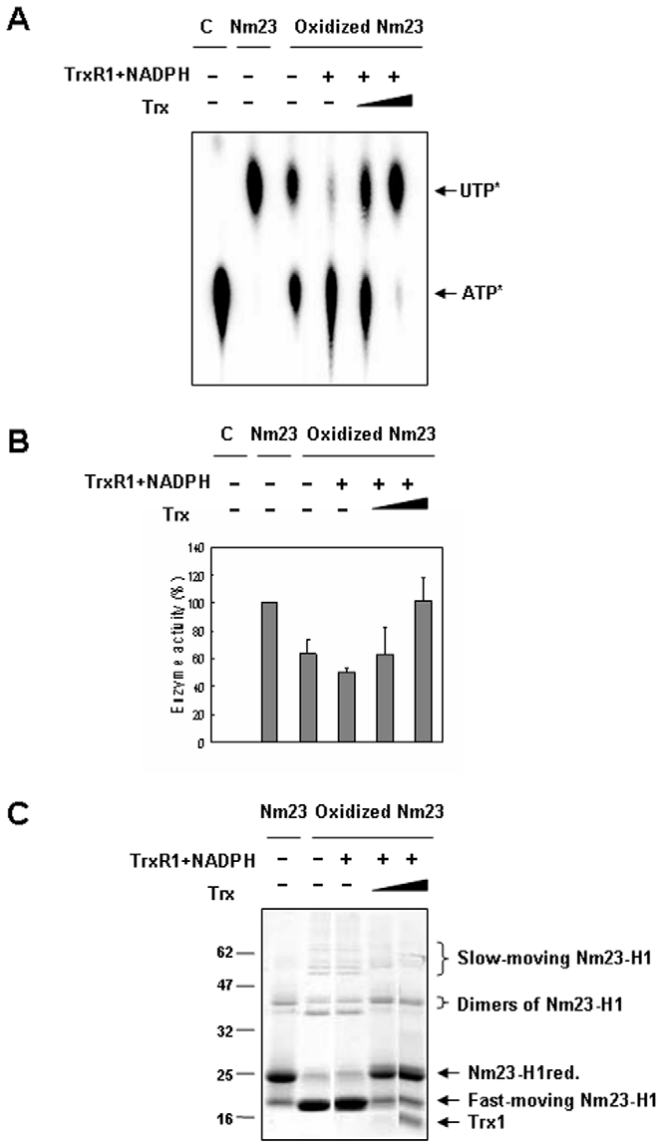
Impaired NDP kinase enzyme activity of oxidized Nm23-H1 is restored by of NADPH-TrxR-Trx system. (A,B) Recombinant Nm23-H1 proteins (100 µM) were oxidized and reincubated with 200 µM NADPH, 80 µM rat TrxR1 and various concentration of Trx (0, 10 and 50 µM) same as in Figure 5. Enzymatic activities were measured on TLC plate (A) and quantitatively analyzed (B). (A) Same samples were separated on % SDS-PAGE under non-reducing condition.

To determine whether the Nm23-H1 reduced from its oxidized form by NADPH-TrxR-Trx system, remains functional, we examined the enzymatic activities of Nm23-H1 after incubating with TrxR1 and NADPH with and without Trx. As shown in Figure 7A and 7B, oxidized Nm23 has low enzymatic activity, but reduction of oxidized Nm23 by incubating it in NADPH-TrxR-Trx system recovered the enzymatic activity in a Trx concentration dependent manner. To examine which oxidized forms of Nm23-H1 are reduced by NADPH-TrxR-Trx system, various populations of oxidized Nm23-H1 protein profiles were analyzed with SDS-PAGE under non-reducing conditions. We found that oxidized Nm23-H1 showed intra- and inter-disulfide bonds, and that intra- and specific inter-dimers of Nm23-H1 were reduced by NADPH-TrxR-Trx system (Figure 7C). These results demonstrate that Nm23-H1 is easily oxidized to inactive forms, which can be readily reduced by NADPH-TrxR-Trx system to regain its cellular functions.

### Oxidation of Nm23-H1 inhibit its metastasis suppressing ability

**Figure 8:**
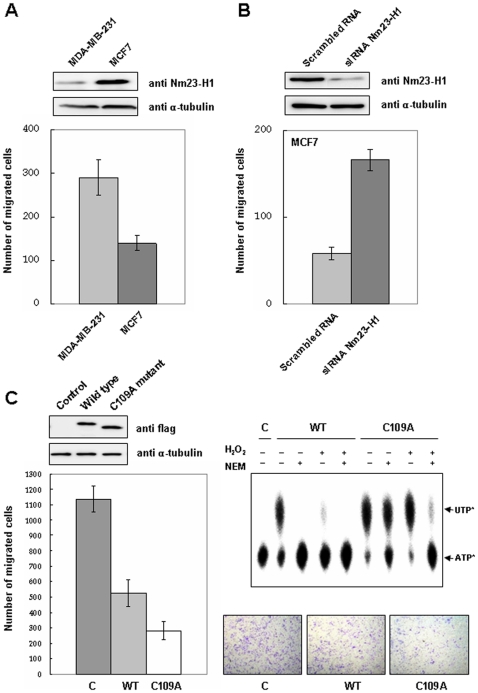
NDP kinase enzymatic activity of Nm23-H1 is required for its ability to suppress metastasis. (A) Comparison of Nm23-H1 expression level and invasion potential between MDA-MB-231, invasive breast cancer cell line, and MCF-7, non invasive breast cancer cell line. Upper panel shows the expression level of Nm23-H1 and α-tubulin as a control, and lower panel, invasive potential in matrigel assay. (B) Comparison of invasion potential of MCF-7 cells reducing Nm23-H1 expression level by siRNA. Upper panel shows the expression level of Nm23-H1 and α-tubulin as a control, and lower panel, invasive potential in matrigel assay. (C) Invasion potential of MDA-MB-231 cells transiently transfected with pFlag-CMV-2, pFlag-Nm23-H1 or pFlag-Nm23-H1 C109A mutant were examined. Western analysis of cell lysates using indicated antibody were employed after separation on 12% SDS-PAGE. Cells overexpressing pFlag-CMV-2, pFlag-Nm23-H1, pFlag-Nm23-H1 C109A mutant, or pFlag-Nm23-H1 H118F mutant were seeded on matrigel-pre-coated upper chamber (3×10^4^ cells/150 µL) and invasion potentials were measured (left panel). The recombinant wild type Nm23-H1 or C109A mutant were treated with 5 mM H_2_O_2_ or 20 mM NEM for 1 h at 37°C, and NDP kinase activity were measured (right panel).

It is well known that metastatic potential for breast cancer cells inversely correlates with their Nm23-H1 content [37]. To determine whether oxidation of cellular Nm23-H1 affects cell migration, we examined the invasion potential of breast cancer cell lines, MDA-MB-231 and MCF-7 cells, transiently transfected with Nm23-H1 C109A mutant. Here, MDA-MB-231, a highly invasive cell line, express lower levels of Nm23-H1 than MCF-7 cells, non-invasive cells (Figure 8A). When we knocked down the Nm23-H1 with siRNA of Nm23-H1 in MCF-7 cells, the invasive potential of MCF-7 significantly increased. These results clearly show the inverse relationship between Nm23-H1 expression level and invasive potential of breast cancer cells.

To investigate the relationship between Nm23-H1's enzymatic activity and its ability to influence cell migration, we examined the invasive potential of cell line, MDA-MB-231 transiently transfected with various Nm23-H1 mutants. We employed wild type Nm23-H1 and C109A mutant which resists oxidative stress, called as active mutant. The enzyme activity of cells transfected with wild Nm23-H1 was completely abolished by oxidative stress and sulfhydryl reagent, N-ethylmaleimide (NEM). On the other hand, transfection of the cells with mutant, C109A, caused no loss in the enzymatic activity under oxidative stress, but some loss of enzymatic activity was detected by combining oxidative stress and NEM treatment because of the inactivation of active site histidine residue by NEM. This indicates that C109A mutant can be used as an active mutant by oxidative stress. MDA-MB-231 cells transiently transfected with various constructs including pFlag-CMV-2, pFlag-Nm23-H1, and pFlag-Nm23-H1 C109A mutants were examined for their invasive potentials by seeding on matrigel-pre-coated upper chamber (3×10^4^ cells/150 µL) and measuring the migrating cells after 24 h. As shown in Figure 8C, overexpression of Nm23-H1 wild type and C109A mutant discernibly reduced the migration, and there was further inhibition in cells overexpressing C109A mutant. This indicates that active Nm23-H1 is required for the inhibition of invasive potential of the cells.

**Figure 9:**
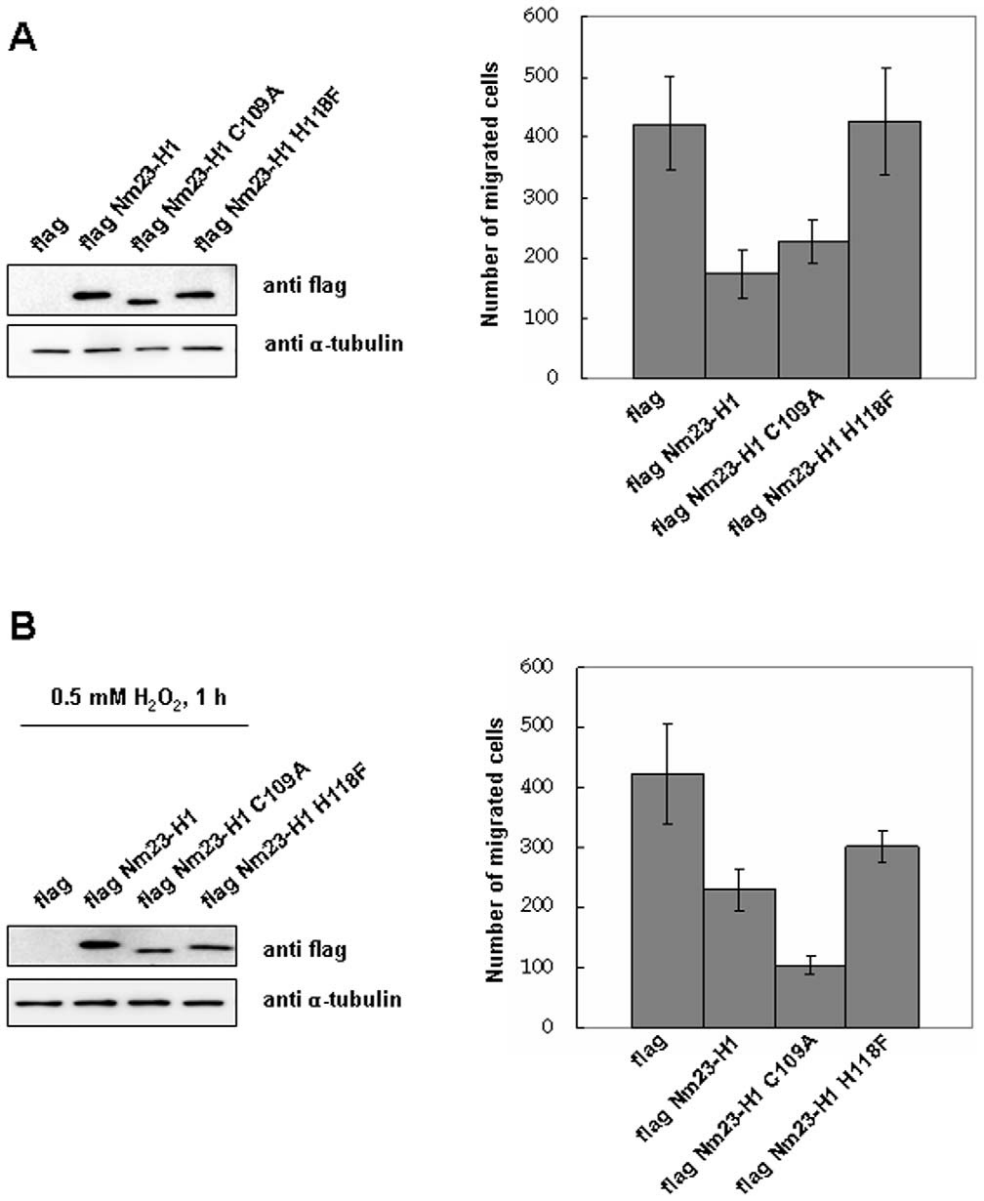
Oxidative stress inhibits the metastasis suppression potential of Nm23-H1. Invasion potential of MDA-MB-231 cells transiently transfected with pFlag-CMV-2, pFlag-Nm23-H1 or pFlag-Nm23-H1 C109A mutant were examined. Western analysis of cell lysates using indicated antibody were employed after separation on 12% SDS-PAGE. Cells overexpressing pFlag-CMV-2, pFlag-Nm23-H1, pFlag-Nm23-H1 C109A mutant or pFlag-Nm23-H1 H118F mutant were exposed to 0.5 mM H_2_O_2_ for 1 h (B) or none (A). And they were seeded on matrigel-pre-coated upper chamber (3×10^4^ cells/150 µL) and invasion potentials were measured.

To examine whether cellular oxidation of Nm23-H1 at C109 regulates their invasive potential, MDA-MB-231 cells transiently transfected with various constructs were exposed to oxidative stress and their migration potentials compared. The migration potentials of MDA-MB-231 cells transiently transfected with various constructs including pFlag-CMV-2, pFlag-Nm23-H1, and pFlag-Nm23-H1 C109A and pFlag-Nm23-H1 H118F mutant were measured with and without treatment with 0.5 mM H_2_O_2_ for 1 h. Under this oxidative stress, wild Nm23-H1 partially lost enzyme activity, and the H118F mutant, the active site mutant, had no enzymatic activity, but C109A mutant retained its normal activity, as did the positive mutant. As expected, invasiveness of cells expressing wild and C109A mutant Nm23-H1 were similarly decreased, compared to cells expressing control and H118F mutant (Figure 9A). However, inhibition of invasiveness was discernibly decreased in cells overexpressing wild Nm23-H1 under oxidative stress, but the invasive ability of cells expressing C109A mutant which is not easily oxidized by H_2_O_2_ treatment, remained inhibited (Figure 9B). This indicates that Nm23-H1 activity is regulated by oxidation of its Cys109, and that it affects the ability of Nm23-H1 as a tumor metastasis suppressor.

## Discussion

Nm23 plays multiple key roles in tumor metastasis, oncogenesis, proliferation, development and differentiation. Song *et al*. suggested that cellular Nm23 is involved in reactive oxygen species (ROS) signaling pathway and that disulfide cross-linking of Nm23 in response to H_2_O_2_ might be a mechanism for regulation of these cellular functions [20]. This study a) confirmed that the biological function of Nm23-H1 is regulated by oxido-reduction; b) demonstrated that Cys109 of cellular Nm23, not in its active site, can be oxidized to cysteic acid, to produce disulfide bond with glutathione and inter- and intra-disulfide bonds with C4, C145 and C109; and that oxidized Nm23-H1 lost its enzymatic activity as well as ability to suppress tumor metastasis; and that oxidized Nm23-H1 interacts with thioredoxin reductase (TrxR) and is reduced by NADPH-TrxR-Trx system. This study is the first to identify the oxidative modifications of Nm23-H1 and propose that cys-109, not in its catalytic center, regulates its biological functions by oxido-reduction mechanisms.

Reactive cysteine residues are readily oxidizable, by cellular ROS to various oxidation forms including sulfenic, sulfinic, sulfonic acid, intra- and inter-disulfide and glutathionylation. Recently, other minor oxidative modifications have been identified employing the proteomic technology combined with modification identification strategies and newly developed algorithms [36]–[38]. We comprehensively examined the modifications of cellular Nm23-H1 using nanoAcquity™ UPLC™/ESI-q-TOF tandem MS and found that Cys109 was variously modified to cysteic acid, or to acquire inter- and intra-disulfide bonds or to become glutathionylated (Figure 1). Cys109 is located in the trimeric interface Kpn loops (residue 99–119) and is involved in contacts between subunits in hexameric structure. Previously, it was shown that interdisulfide Cys109-Cys109 formation affects the enzymatic activity of Nm23-H1 and its oligomeric structure [20]. In this study, we demonstrated that glutathionylation of Cys109 in Nm23-H1 by H_2_O_2_ also inactivates enzymatic activity by damaging the binding of the nucleoside in active site (Figure 4). Reversible S-glutathionylation is an important post-translational modification, protecting protein cysteines from irreversible oxidation and serving to transducer redox signals. Many proteins including p53 are known to be glutathionylated and regulated by redox [39], [40]. It is known that human p53 is inhibited by glutathionylation of cysteine present in the proximal DNA-binding domain during oxidative stress [39]. These findings suggest that Cys109 plays a key role in cellular response to oxidative stress via glutathionylation as well as disulfide formation.

We demonstrated that oxidized Nm23-H1 can specifically interact with thioredoxin reductase 1 (TrxR1) (Figure 5), which known as a selenium containing pyridine nucleotide disulfide oxidoreductase catalyzing the NADPH-dependent reduction of the redox protein thioredoxin, as well as of other endogenous and exogenous compounds [23]. It is necessary to examine whether Nm23 cellular activity is regulated by NADPH-TrxR-Trx system. Thiol reducing systems including Trx, TrxR, and NADPH, reduce oxidized cysteine groups on protein through interaction with the redox-active center of Trx (CGPC, Trx-(SH)_2_) to form a disulfide bond (Trx-S2), which can be reduced by TrxR and NADPH. Trxs are family of protein having well conserved catalytic site (WCGPCK) through evolution [41]. Trx is a major reducing system that maintains the redox balance of the cell by reducing the various oxidized proteins by ROS. Here we showed that oxidized Nm23-H1 is a substrate of NADPH-TrxR-Trx oxidoreduction system coupled with reduced Trx. (Figure 6). NDP kinase activity was abolished on treatment with H_2_O_2_, and was recovered by NADPH-TrxR-Trx system in a Trx dose-dependent manner. We then examined whether oxidized Nm23-H1 is reduced by NADPH-TrxR-Trx system. We observed oxidized Nm23-H1 on non-reducing gel electrophoresis by incubating various combinations of NADPH-TrxR-Trx–Nm23 components. As shown in Figure 7C, reduced Nm23-H1, on treatment with H_2_O_2_ can induce the formation of various oxidation products: fast moving intra-molecular cross-linked Nm23 and dimers of intermolecular from cross linking; these oxidized Nm23-H1 can be reduced to monomers by increasing the amount of Trx in NADPH-TrxR-Trx system. Especially, the fast-moving intra-molecular cross-linked Nm23-H1 and one of the dimers can be easily reduced by this reduction system. However, one of the oxidized dimers was not reduced by TrxR-Trx system. This indicates that NADPH-TrxR-Trx system reduces only some specific oxidation forms.

The relationship between Nm23-H1 expression level and cell migration capability was examined in two breast cancer cell lines, the invasive MDA-MB-231 cell line and the non-invasive MCF-7 cell line. High expression of Nm23-H1 decreased cell migration capability, and reduction of Nm23-H1 by RNAi treatment reversed this effect (Figure 7). NDP kinase activity of Nm23-H1 appears to be critical for its biological effects. For example, in contrast to wild-type Nm23 which promotes neurite outgrowth, a point mutant of Nm23 (H118A) that lacks NDP kinase activity does not promote neurite outgrowth, but actually suppresses nerve growth factor-induced neurite outgrowth [42], [43]. Also, the other hand, no correlation of NDP kinase activity and metastasis suppression was reported in previous work [44], [45]. In addition, Nm23-H2 can bind to a nuclease hypersensitive element on the human *c-myc* promoter and transactivate gene expression *in vitro and in vivo* [10], and this transactivation is independent of NDP kinase activity [46], and an enzymatically inactive mutant Nm23-H118F was found to inhibit progesterone-induced maturation [47]. Thus, some functions of Nm23 appear to be independent of its NDPK activity. In this study, we examined the metastatic potential of MDA-MB-231 expressing various Nm23-H1 mutants, and found that oxidation of Nm23-H1 inhibits the metastasis suppression and that the C109A mutant, not oxidized by ROS, was not affected by oxidative stress. These findings suggest that oxidative modifications of Nm23 regulate the functions of Nm23-H1, NDP kinase activity, and cell migration potential. Regulation of migration potential by Nm23-H1 involves either change in NDP kinase enzymatic activity or in oligomeric structure. A previous report showed that the oligomeric interactions play a major role in the stability of catalytic domain and regulate various cellular functions [48]. Recently, protein interaction changes in oxidized GAPDH were demonstrated [49], and indicate that oxidation status of proteins are important to regulate the activity and structure.

In summary, this study shows for the first time that Nm23-H1 is regulated by oxidoreduction of Cys109; that glutathionylation and disulfide formation by ROS inhibit the NDPK activity and metastasis suppressor ability of Nm23-H1. And disulfide formation is easily reduced by NADPH-TrxR-Trx system. Further studies are needed, directed at identifying the elements involved in the regulation of each oxidized form, in order to understand the molecular mechanisms by which Nm23-H1 acts as a suppressor of metastasis. Also we are in progress for further study to examine whether isoform Nm23-H2 function, known as transcriptional activator of c-myc gene, is regulated by oxido-reduction system.

## Supporting Information

Figure S1 Quantitative analysis of PTM peptides were performed using precursor ion intensities. Each mass chromatogram was narrowly extracted, integrated, and summarized.
(0.22 MB TIF)

Figure S2 The CID fragments of glutathionylated “GDFCIQVGR” peptide were predicted using MASS FRAGMENT version 2.0 software (Waters Co. UK).
(0.35 MB TIF)

Figure S3 NADPH-TrxR-Trx assay were performed as same way as in Figure 6, by monitoring the absorbance change at 340 nm. Absorbance decreases by conversion of NADPH to NADP were measured as TR activity in various combinations of substrates. It turns out that oxidized Nm23-H1 is a substrate of TrxR-Trx-NADPH system.
(0.22 MB TIF)
