# Supplementary figures and images for: Multiple Functions of Nm23-H1 Are Regulated by Oxido-Reduction System
Source: PLoS One. 2009 Nov 23;4(11):e7949. doi: 10.1371/journal.pone.0007949 (PMC2776532; doi:10.1371/journal.pone.0007949)

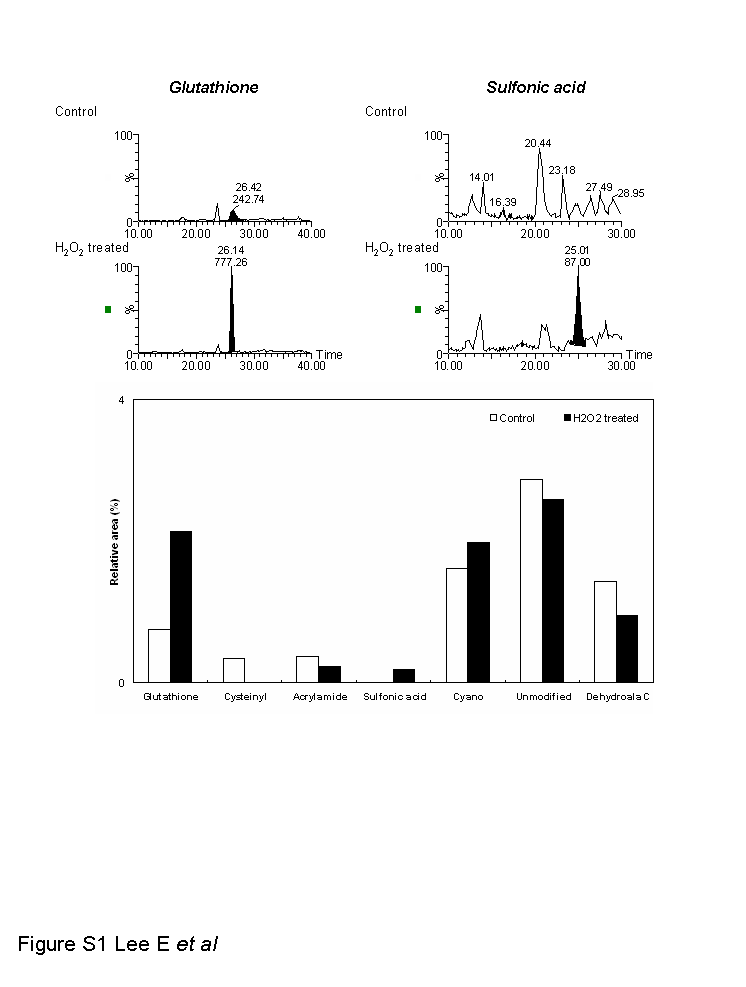

Supplement: Figure S1 — Quantitative analysis of PTM peptides were performed using precursor ion intensities. Each mass chromatogram was narrowly extracted, integrated, and summarized. (0.22 MB TIF) [file pone.0007949.s001.tif]

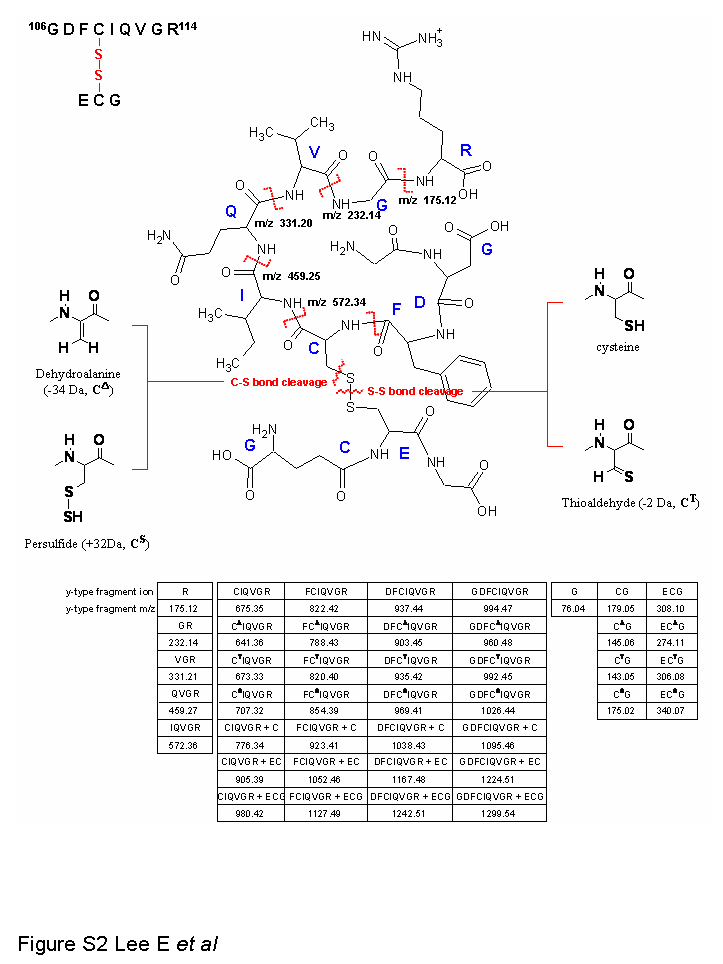

Supplement: Figure S2 — The CID fragments of glutathionylated “GDFCIQVGR” peptide were predicted using MASS FRAGMENT version 2.0 software (Waters Co. UK). (0.35 MB TIF) [file pone.0007949.s002.tif]

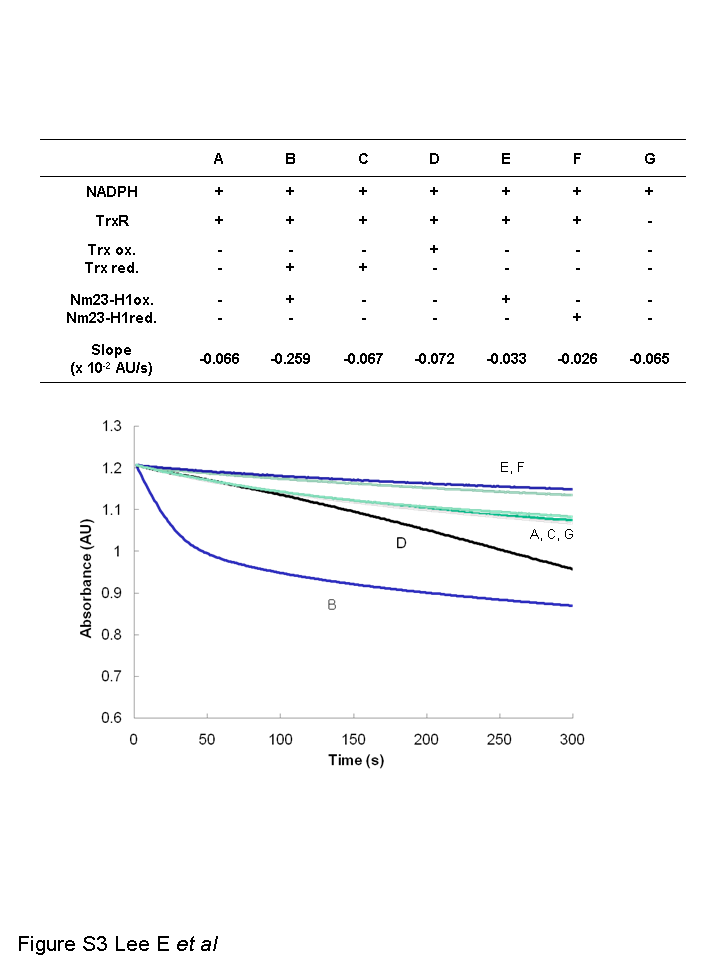

Supplement: Figure S3 — NADPH-TrxR-Trx assay were performed as same way as in Figure 6, by monitoring the absorbance change at 340 nm. Absorbance decreases by conversion of NADPH to NADP were measured as TR activity in various combinations of substrates. It turns out that oxidized Nm23-H1 is a substrate of TrxR-Trx-NADPH system. (0.22 MB TIF) [file pone.0007949.s003.tif]
